# An association study of m6A methylation with major depressive disorder

**DOI:** 10.1186/s12888-024-05760-w

**Published:** 2024-05-07

**Authors:** Ying Li, Peidong Miao, Fang Li, Jinsong Huang, Lijun Fan, Qiaoling Chen, Yunan Zhang, Feng Yan, Yan Gao

**Affiliations:** 1 Dalian Seventh People’s Hospital, No. 179 Lingshui Road, Ganjingzi District, Dalian City, Liaoning Province PR China; 2 Dalian No. 3 People’s Hospital, Department of Interventional Radiology, Dalian, PR China

**Keywords:** Major depressive disorder(MDD), N6-methyladenosine (m6A) genes, ELAVL1 gene, YTHDC2 gene

## Abstract

**Objective:**

To find the relationship between N6-methyladenosine (m6A) genes and Major Depressive Disorder (MDD).

**Methods:**

Differential expression of m6A associated genes between normal and MDD samples was initially identified. Subsequent analysis was conducted on the functions of these genes and the pathways they may affect. A diagnostic model was constructed using the expression matrix of these differential genes, and visualized using a nomogram. Simultaneously, an unsupervised classification method was employed to classify all patients based on the expression of these m6A associated genes. Following this, common differential genes among different clusters were computed. By analyzing the functions of the common differential expressed genes among clusters, the role of m6A-related genes in the pathogenesis of MDD patients was elucidated.

**Results:**

Differential expression was observed in ELAVL1 and YTHDC2 between the MDD group and the control group. ELAVL1 was associated with comorbid anxiety in MDD patients. A linear regression model based on these two genes could accurately predict whether patients in the GSE98793 dataset had MDD and could provide a net benefit for clinical decision-making. Based on the expression matrix of ELAVL1 and YTHDC2, MDD patients were classified into three clusters. Among these clusters, there were 937 common differential genes. Enrichment analysis was also performed on these genes. The ssGSEA method was applied to predict the content of 23 immune cells in the GSE98793 dataset samples. The relationship between these immune cells and ELAVL1, YTHDC2, and different clusters was analyzed.

**Conclusion:**

Among all the m6A genes, ELAVL1 and YTHDC2 are closely associated with MDD, ELAVL1 is related to comorbid anxiety in MDD. ELAVL1 and YTHDC2 have opposite associations with immune cells in MDD.

**Supplementary Information:**

The online version contains supplementary material available at 10.1186/s12888-024-05760-w.

## Introduction

According to the report of the World Health Organization, there are about 322 million patients with MDD worldwide, and the number is increasing year by year (https://www.who.int/news-room/fact-sheets/detail/depression). MDD is a complex multifactorial disease, constituting one of the foremost social burdens worldwide [1], yet its pathogenesis remains unclear. Therefore, investigating the pathogenesis of depression can enhance diagnostic accuracy and alleviate the medical burden.

It is reported that MDD is caused by the combined effect of genetic factors and environmental stress [2]. The mechanism by which nucleic acids undergo chemical modifications to respond to stress stimuli and environmental factors is termed epigenetics [3]. It focuses on the heritable changes in gene expression without alterations in DNA sequence, ultimately leading to phenotypic changes. Epigenetic mechanisms include DNA methylation, RNA methylation, histone acetylation and methylation, miRNA regulation, among others.

In recent years, the relationship between RNA methylation in epigenetics and MDD has attracted people’s attention. Over 100 different types of RNA modifications have been discovered in eukaryotes, among which N6-methyladenosine (m6A) is the most abundant in RNA methylation [4]. Studies have found that dynamic changes in m6A modification levels can directly impact RNA metabolism and protein function, and regulate circadian rhythms, neurogenesis, and brain development in organisms [5]. Alterations in the m6A profile are associated with various diseases, including cancer and psychiatric disorders [6]. Research indicates that m6A plays a significant role in stress-related psychiatric disorders, including major depressive disorder [7, 8].

The purpose of this study is to investigate the relationship between m6A methylation-related genes and depression by bioinformatics analysis.

## Methods

### Data acquisition

The dataset analyzed in this study was obtained from the GEO (Gene Expression Omnibus) database, specifically from the GSE98793 dataset [9]. This dataset comprises whole-blood samples from 128 individuals diagnosed with severe depression (of which 64 were diagnosed with comorbid generalized anxiety disorder) and 64 healthy controls. Transcriptional profiling was performed using the GPL570 platform ([HG-U133_Plus_2] Affymetrix Human Genome U133 Plus 2.0 Array).

### Analysis platform

**Fig. 1:**
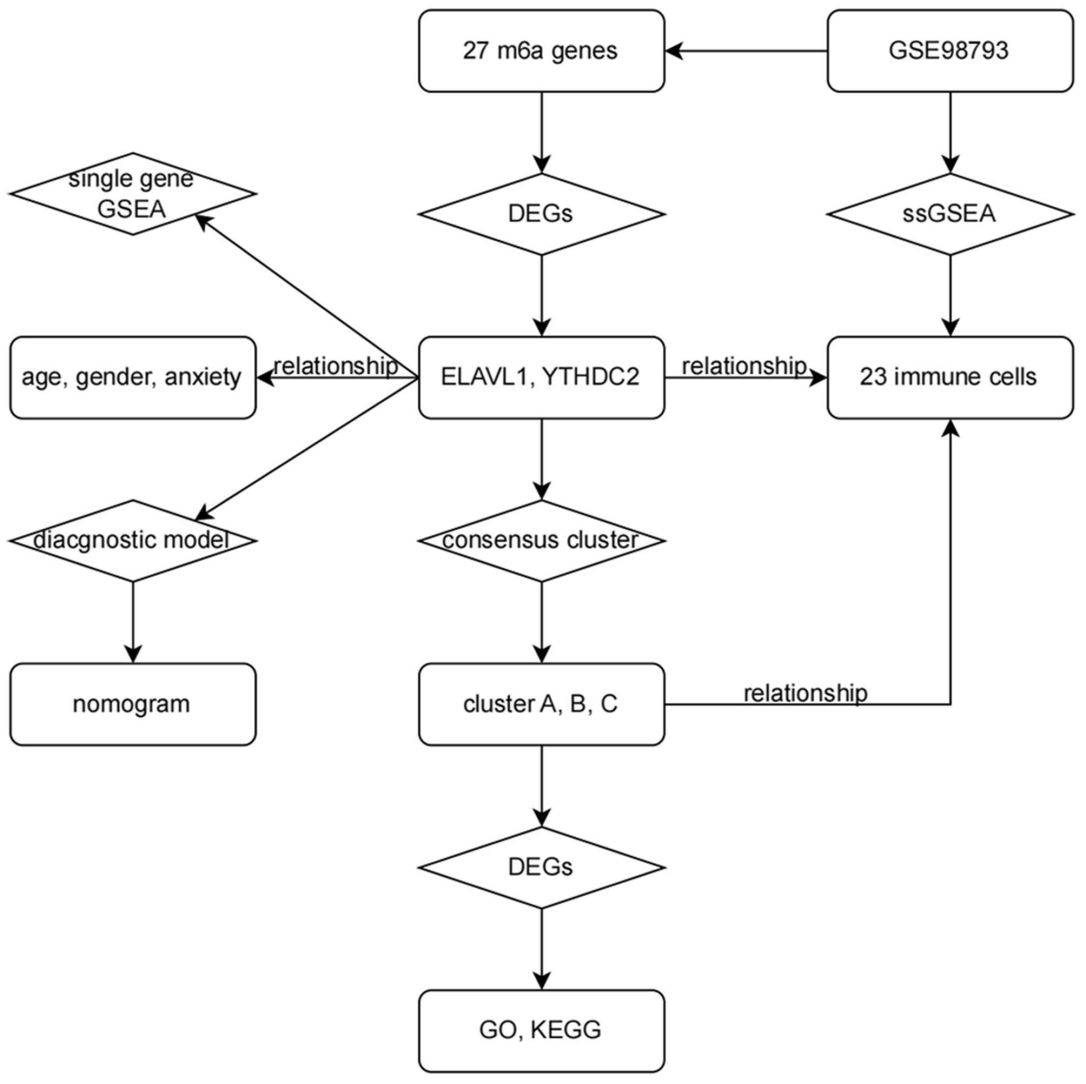
Roadmap of our study

The analyses were conducted using R language (Version: 4.3.0) within Rstudio (Version: 2023.12.1 + 402). The main workflow of this study is illustrated in Fig. 1.

### Differential expression of m6A associated genes

The expression matrix of the GSE98793 dataset was downloaded from GEO database (https://www.ncbi.nlm.nih.gov/geo/query/acc.cgi?acc=GSE98793), and the expression matrix of m6A-related genes reported in literature was extracted [10]. Among these 27 genes, 9 are writers (METTL3, METTL14, METTL16, WTAP, VIRMA, ZC3H13, RBM15, RBM15B, CBLL1), 15 are readers (YTHDC1, YTHDC2, YTHDF1, YTHDF2, YTHDF3, HNRNPC, FMR1, LRPPRC, HNRNPA2B1, IGFBP1, IGFBP2, IGFBP3, RBMX, ELAVL1, IGF2BP1), and the remaining 2 are erasers (FTO, ALKBH5). Differential expression analysis between the disease and control groups was conducted using the wilcox test (*P* < 0.05). Subsequently, the relationship between the expression levels of these differentially expressed m6a associated genes and clinical phenotypes (age, gender, presence of anxiety) provided by GSE98793 dataset was analyzed. Finally, the functional implications of these genes were explored using the single-gene Gene Set Enrichment Analysis (GSEA) method implemented in the corTest function of the psych R package, revealing potential pathways affected by these genes [11].

### Nomogram construction and diagnostic model establishment

Based on the identified differentially expressed m6A associated genes, a linear regression model predicting Major Depressive Disorder (MDD) was established using the rms package in R [12]. A nomogram was generated to visualize the diagnostic model. Calibration curves and Decision Curve Analysis (DCA) curves were plotted to assess the accuracy and utility of the model.

### Clustering analysis of differentially expressed m6A associated genes

Additionally, utilizing the expression matrix of differentially expressed m6A associated genes, unsupervised clustering analysis (consensus clustering) was performed on the 128 MDD patients using the ConsensusClusterPlus package in R [13].

### Differential gene expression between clusters

Differential expression analysis between different clusters was conducted by comparing the expression profiles of all genes using the limma package [14]. Criteria for differential expression were set as logFC > = 0.2 and adjusted *p*-value < 0.05. These differentially expressed genes are closely associated with m6A associated genes. Further functional analysis of these genes aims to elucidate how m6A associated genes influence biological changes in MDD patients.

### ssGSEA prediction of immune cell composition in patients

Subsequently, single-sample Gene Set Enrichment Analysis (ssGSEA) was employed to predict the composition of immune cells in peripheral blood of all MDD patients in the GSE98793 dataset. The analysis was conducted using the GSEABase and GSVA packages in R [15, 16]. A reference gene set comprising 23 immune cell types obtained from literature was utilized [17]. These 23 immune cell types are as follows: Activated B cell, Activated CD4 T cell, Activated CD8 T cell, Activated dendritic cell, CD56 bright natural killer cell, CD56 dim natural killer cell, Eosinophil, Gamma delta T cell, Immature B cell, Immature dendritic cell, Myeloid-derived suppressor cell (MDSC), Macrophage, Mast cell, Monocyte, Natural killer T cell, Natural killer cell, Neutrophil, Plasmacytoid dendritic cell, Regulatory T cell, T follicular helper cell, Type 1 T helper cell, Type 17 T helper cell, and Type 2 T helper cell. Differences in immune cell composition between different clusters were assessed (*p* < 0.05), along with the relationship between expression levels of differentially expressed m6A associated genes and immune cell abundance.

## Results

### Differential expression of m6A associated genes

**Fig. 2:**
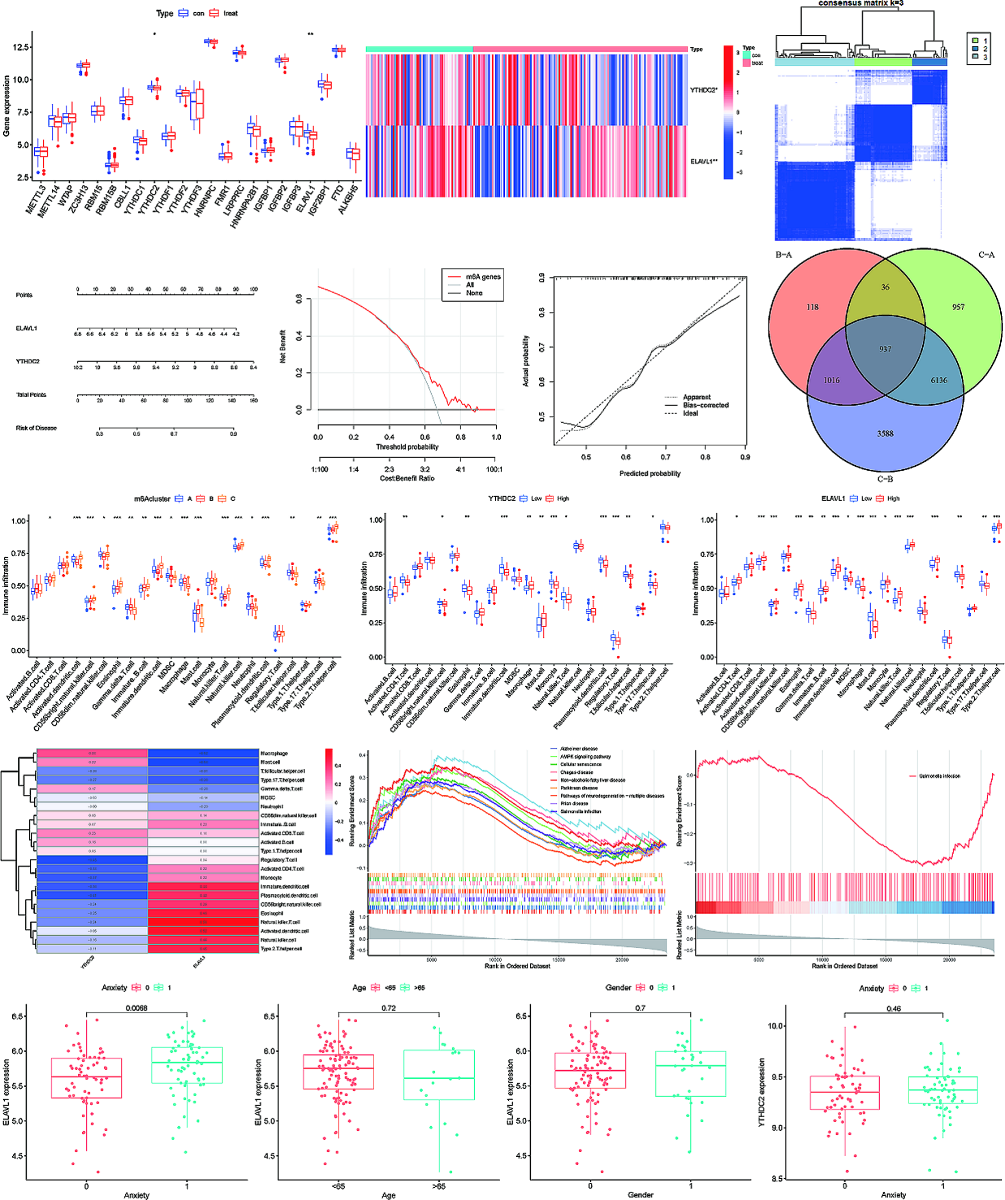
(**A**) Boxplots depicting the expression levels of 23 m6A-related genes. Blue represents the healthy population, while red represents MDD patients. It can be observed that there are differences in the expression of YTHDC2 and ELAVL1 between groups, with higher expression levels in the healthy population compared to MDD patients. (**B**) Heatmap illustrating the expression levels of YTHDC2 and ELAVL1 in the healthy population (con) and MDD patients. (**C**) The ConsensusClusterPlus package in R divided all MDD patients into 3 clusters. (**D**) Nomogram visualizing the linear regression model established based on the expression levels of ELAVL1 and YTHDC2. (**E**, **F**) Clinical decision curves and consistency curves of the m6A-related gene model. (**G**) Venn diagram depicting the intersection of differentially expressed genes among the 3 clusters. (**H**, **I**, **J**) Predicted immune cell abundance by ssGSEA. Boxplots showing differences in immune cell abundance among the 3 clusters (**H**), between high and low expression groups of YTHDC2 (**I**), and between high and low expression groups of ELAVL1 (**J**). (**K**) Heatmap illustrating the correlation between YTHDC2 and ELAVL1 expression and the abundance of 23 immune cell types. (**L**) Single-gene GSEA results for ELAVL1. (**M**) Single-gene GSEA results for YTHDC2. (**N**, **O**, **P**) Boxplots showing the relationship between ELAVL1 expression and the presence of anxiety (**N**), age (**O**), and gender (**P**). (**Q**) Boxplot illustrating the relationship between YTHDC2 expression and the presence of anxiety

In our analysis of the 27 m6A associated genes, we observed differential expression of ELAVL1 and YTHDC2 between the MDD and control groups (Fig. 2A, B). Both genes encode methylated RNA-binding proteins capable of recognizing and binding to m6A modification sites on RNA, thereby initiating downstream signaling transduction or regulating RNA metabolism processes. Their expression levels were both lower in MDD patients compared to the control group (Fig. 2A). Within the samples included in the GSE98793 dataset, ELAVL1 expression was associated with comorbid anxiety in MDD patients (*P* < 0.05) (Fig. 2N) but showed no correlation with patient age or gender (*P* > 0.05) (Fig. 2O, P). YTHDC2, on the other hand, exhibited no association with age, gender, or comorbid anxiety (*P* > 0.05) (Fig. S.1 A, B, Fig. 2Q). The single-gene GSEA results for ELAVL1 in this dataset (*P* < 0.05) included enrichment in KEGG pathways such as Non-alcoholic fatty liver disease, Alzheimer’s disease, Prion disease, Pathways of neurodegeneration - multiple diseases, Salmonella infection, AMPK signaling pathway, Cellular senescence, Adipocytokine signaling pathway, Chagas disease, Human papillomavirus infection, Parkinson’s disease, MAPK signaling pathway, Hypertrophic cardiomyopathy, ECM-receptor interaction, Glucagon signaling pathway, Insulin resistance, Human immunodeficiency virus 1 infection, TNF signaling pathway, Epstein-Barr virus infection, and Autophagy - animal (Fig. 2L). In contrast, YTHDC2’s single-gene GSEA results were enriched only in the Salmonella infection KEGG pathway (*P* < 0.05) (Fig. 2H).

### Linear regression model and nomogram

A linear regression model was established based on the expression levels of ELAVL1 and YTHDC2, and visualized using a nomogram (Fig. 2D). From the calibration curve, it is evident that this model can accurately predict whether samples in the GSE98793 dataset have Major Depressive Disorder (MDD) (Fig. 2F). Furthermore, the Decision Curve Analysis (DCA) curve indicates that the application of this model can lead to a higher net benefit for clinical decision-making (Fig. 2E).

### Unsupervised clustering

Utilizing the expression matrix of ELAVL1 and YTHDC2, the ConsensusClusterPlus package in R divided all MDD patients into 3 clusters (Fig. 2C) [13]. By comparing the differential gene expression between these three clusters, a total of 937 genes were found to be significantly differentially expressed across the three clusters after taking the intersection (Fig. 2G).

### Enrichment analysis

In the enrichment analysis, we found that differentially expressed genes were enriched in 7 Gene Ontology Biological Process (GO: BP) terms (filtering criteria: *p*-value < 0.05 and q-value < 0.05). These terms include regulation of viral process, viral process, positive regulation of viral genome replication, positive regulation of viral process, movement in host, biological process involved in interaction with host, and viral life cycle (Tab. S1). However, no enrichment was observed in KEGG pathways under the filtering criteria of *p*-value < 0.05 and q-value < 0.05.

### ssGSEA analysis

The ssGSEA analysis predicted the levels of 23 immune cell types in the peripheral blood of 128 MDD patients. Except for Activated B cells, Activated CD8 T cells, Monocytes, Regulatory T cells, and Type 1 T helper cells, the expression levels of the remaining 18 immune cell types varied significantly between different clusters (Fig. 2H).

Interestingly, in the correlation analysis, we found that the correlation between YTHDC2 and ELAVL1 with the 23 types of immune cells was nearly precisely opposite (Fig. 2K). For instance, the expression level of ELAVL1 was positively correlated with the abundance of Regulatory T cells, Activated CD4 T cells, Monocytes, Immature dendritic cells, Plasmacytoid dendritic cells, CD56 bright natural killer cells, Eosinophils, Natural killer T cells, Activated dendritic cells, Natural killer cells, and Type 2 T helper cells. Conversely, the expression level of YTHDC2 was negatively correlated with these cell types. Additionally, the expression level of ELAVL1 showed a significant negative correlation (correlation coefficient <-0.5) with the abundance of Macrophages and Mast cells, while YTHDC2 exhibited a positive correlation with these cell types. The two genes has opposite correlations with immune cell abundance raise.

Furthermore, we conducted analysis using two box plots to examine whether there were differences in the levels of the 23 immune cell types between high and low expression groups of YTHDC2 and ELAVL1 within MDD patients (*P* < 0.05) (Fig. 2I, J).

In the comparison between high and low expression groups of ELAVL1, significant differences (*p* < 0.05) were observed in the levels of Activated CD4 T cells, Activated dendritic cells, CD56 bright natural killer cells, Eosinophils, Gamma delta T cells, Immature B cells, Immature dendritic cells, Myeloid-derived suppressor cells (MDSCs), Macrophages, Mast cells, Monocytes, Natural killer T cells, Natural killer cells, Plasmacytoid dendritic cells, T follicular helper cells, Type 17 T helper cells, and Type 2 T helper cells. Among these, Gamma delta T cells, MDSCs, Macrophages, Mast cells, T follicular helper cells, and Type 17 T helper cells exhibited a negative correlation with ELAVL1 expression (i.e., higher ELAVL1 expression group had lower immune cell abundance), while the remaining immune cell types showed positive correlations (i.e., higher ELAVL1 expression group had higher immune cell abundance).

Similarly, in the comparison between high and low expression groups of YTHDC2, significant differences (*p* < 0.05) were observed in the levels of Activated CD4 T cells, CD56 bright natural killer cells, Eosinophils, Immature dendritic cells, Macrophages, Mast cells, Monocytes, Natural killer T cells, Plasmacytoid dendritic cells, Regulatory T cells, T follicular helper cells, and Type 17 T helper cells. Among these, only Macrophages and Mast cells exhibited a positive correlation with YTHDC2 expression (i.e., higher YTHDC2 expression group had higher immune cell abundance), while the remaining cell types showed negative correlations (i.e., higher YTHDC2 expression group had lower immune cell abundance).

## Discussion

Depression, as a kind of mental disorder, has been gradually known to people. It is the main cause of disability for patients worldwide, and the probability of suicide is 30 times higher than that of normal people. Since the outbreak of COVID-19 at the end of 2019, the prevalence of depression and anxiety among the public has increased significantly [18], and the proportion of depression and anxiety symptoms among front-line doctors is much higher than that of other health care workers [19]. Depression also imposes a huge financial burden on sufferers, their families and society as a whole. According to the World Health Organization, the loss of productivity due to depression and anxiety will cost the global economy up to one trillion US dollars in 2020, and this loss is still increasing (https://www.who.int/health-topics/depression/). Experts estimate that depression will become a major contributor to the global burden of disease by 2030 [20].

Methylation of m6A refers to a methylation that occurs under the action of adenine 6th N-methyltransferase complex (MTC) on mRNA. The modification level is regulated by methyltransferase and demethyltransferase. RNA binding proteins affect RNA metabolism. The abundance of m6A modification in brain tissue is higher than that in other tissues [21], and the m6A spectrum in the human brain shows that genes containing brain-specific m6A are enriched in synapses and neuronal pathways [22]. m6A modification plays a crucial role in different stages of brain neurodevelopment and also maintains the normal function of the nervous system. Multiple studies have shown that it affects nervous system development, learning ability, memory function, nerve regeneration, synaptic function, neuron apoptosis, cell proliferation and differentiation [23–27]. Some of them have been confirmed in neurological diseases such as Alzheimer’s disease [28, 29] and Parkinson’s disease [30]. At present, some studies have revealed that some genes regulating m6A are related to depression [7, 31], but the results are limited, the data amount is small, and the specific mechanism is not clear.

The results of this study showed that there were significant differences in the expression levels of m6A related genes ELAVL1 and YTHDC2 in MDD patients and healthy people, which confirmed that ELAVL1 and YTHDC2 were closely related to MDD.

### ELAVL1

The protein encoded by the ELAVL1 gene is a member of the ELAVL family of RNA-binding proteins, which contains several RNA recognition motifs selectively binding to the 3’ untranslated regions of mRNAs, thereby increasing their stability [32–35]. It has been demonstrated that ELAVL1 can bind to m6A-containing mRNAs and promote the stability of MYC mRNA by binding to MYC mRNA containing m6A [36].

The results of this study confirm the association between ELAVL1 expression and comorbid anxiety in MDD patients. Currently, no relevant reports have been found, and the correlation requires further experimental validation, while its specific mechanisms need further research for clarification. The single-gene enrichment analysis of ELAVL1 in MDD patients in this study showed enrichment in pathways such as Alzheimer’s disease, neurodegenerative disease pathways, Parkinson’s disease, and cellular senescence. Previous studies have found that ELAVL1 is involved in the pathogenesis of Parkinson’s disease [37, 38], indicating the involvement of the ELAVL1 gene in the biological mechanism of neurodegeneration in MDD patients during m6A methylation. This study also found enrichment of the ELAVL1 gene in pathways such as the AMPK signaling pathway and the MAPK signaling pathway. Previous research has shown that ELAVL1 is involved in the PI3K-PDK1 pathway [39], which is associated with depression, suggesting that one of the mechanisms by which ELAVL1 is related to MDD is through cellular signal transduction pathways. Additionally, in this study, the ELAVL1 gene was found to be enriched in pathways such as human immunodeficiency virus type 1 infection, Epstein-Barr virus infection pathway, human papillomavirus infection, measles, and Salmonella infection. Previous studies have found that the ELAVL1 gene is associated with susceptibility to human coronavirus infection [40], indicating the biological mechanism of m6A methylation mediated by the ELAVL1 gene in the process of virus infection in MDD patients. The ELAVL1 gene in MDD patients was also found to be enriched in pathways such as cellular senescence, adipocytokine signaling pathway, glucagon signaling pathway, and insulin resistance, indicating its association with metabolic disorders in depressive patients.

### YTHDC2

YTHDC2 belongs to the YTH family members and has been shown to promote translation by resolving secondary structures, facilitate mRNA degradation by interacting with exonucleases, and disrupt the stability of target transcripts by recruiting the decapping enzyme complex [41–43]. YTHDC2 specifically recognizes and binds to m6A-containing RNA, functioning in RNA processing efficiency and stability [44, 45].

It is known that YTHDF2 stabilizes the expression of inflammation-related transcription factors, activates the MAPK and NFkB signaling pathways, and participates in the inflammatory response of macrophages [46]. It participates in biological pathways (go: 004482935): positive regulation of host involvement in viral genome replication [47]. Previous studies have found that YTHDC2 is involved in identifying the structure domains of SARS-CoV-2 proteins and the sequence-based interactions with human peptide ligands, accurately targeting antiviral sites [48]. Previous research has indicated that m6A plays a role in promoting and inhibiting inflammation in the brain [49]. Although it is unclear how m6A mediates inflammatory responses in the context of MDD, it has been found that m6A mediates inflammatory responses in brain diseases such as stroke [50]. The single-gene GSEA results of this study show enrichment of YTHDC2 in the Salmonella infection pathway. These results collectively suggest the involvement of YTHDC2 in virus infection-related biological mechanisms during m6A methylation in MDD patients.

### ELAVL1 and YTHDC2 in virus infection

This study applied artificial intelligence and found that MDD patients could be divided into three groups according to the expression levels of ELAVL1 and YTHDC2 genes. Enrichment analysis of differentially expressed genes between the three groups revealed enrichment in seven biological processes, including virus process regulation, virus processes, positive regulation of viral genome replication, positive regulation of virus processes, intracellular transport, processes involving interaction with the host, and virus life cycle. All of these processes are related to virus infection pathways. Therefore, it is evident that ELAVL1 and YTHDC2 genes may be associated with the m6A methylation process in MDD through virus infection pathways.

### Correlation of ELAVL1 and YTHDC2 with immune cells

Previous studies have shown that MDD is closely related to the regulation of the immune system [51–53], and it is known that the immune cells of the three groups are significantly different from each other through calculation, which confirms that it is reasonable to divide MDD patients into three groups. Meanwhile, it can be speculated that the immune system plays an important role in the relationship between m6A methylation and MDD.

Through calculation, this study showed that the immune cell content of individuals with different expression levels of ELAVL1 and YTHDC2 was significantly different, which confirmed that m6A methylation related genes ELAVL1 and YTHDC2 were closely related to the immune cell content of MDD patients. This study found that the correlation of ELAVL1 and YTHDC2 with 23 types of immune cells in MDD patients is almost completely opposite. Although both genes belong to methylated RNA-binding proteins and have similar biological functions (readers) in the methylation process, the inverse correlation with immune cell abundance raises interesting questions about potential causes and biological significance. Further experiments are needed to validate these findings, and their specific mechanisms require further research for clarification.

### Limitations of the study

This study has several limitations. Firstly, the model was constructed using only one dataset without external validation, which inevitably increases the risk of overfitting. Additionally, as is inherent in studies of bioinformatics analysis, our results may not fully represent the true biological context within organisms. Therefore, further biological experiments are necessary to validate the conclusions drawn from this research.

### Electronic supplementary material

Below is the link to the electronic supplementary material.

**Supplementary Material 1**: Box plots of YTHDC2 expression and GO results.

## Data Availability

The data that support the findings of this study are openly available in Gene Expression Omnibus database[GEO: GSE98793](https://www.ncbi.nlm.nih.gov/geo/query/acc.cgi?acc=GSE98793).

## Abbreviations

MDD: Major Depressive Disorder
m6A: N6-methyladenosine
mRNA: Messenger RNA
GO: Gene ontology
KEGG: Kyoto Encyclopedia of Genes and Genomes
GSEA: Gene Set Enrichment Analysis
