# Supplementary material for: An association study of m6A methylation with major depressive disorder
Source: BMC Psychiatry. 2024 May 7;24:342. doi: 10.1186/s12888-024-05760-w (PMC11075325; doi:10.1186/s12888-024-05760-w)
Supplement: Supplementary file 1 — Supplementary Material 1: Box plots of YTHDC2 expression and GO results. [file 12888_2024_5760_MOESM1_ESM.docx]

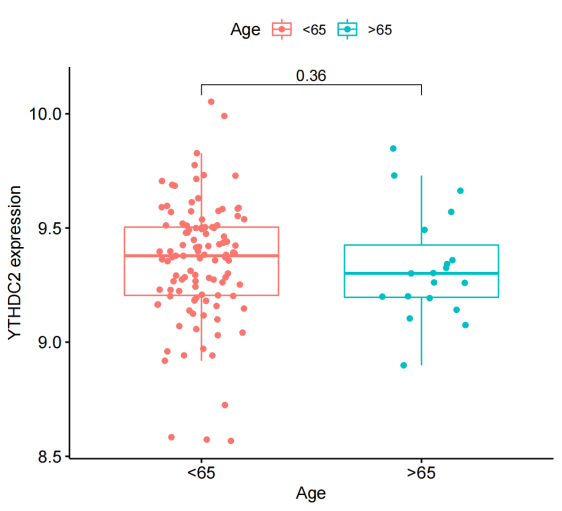

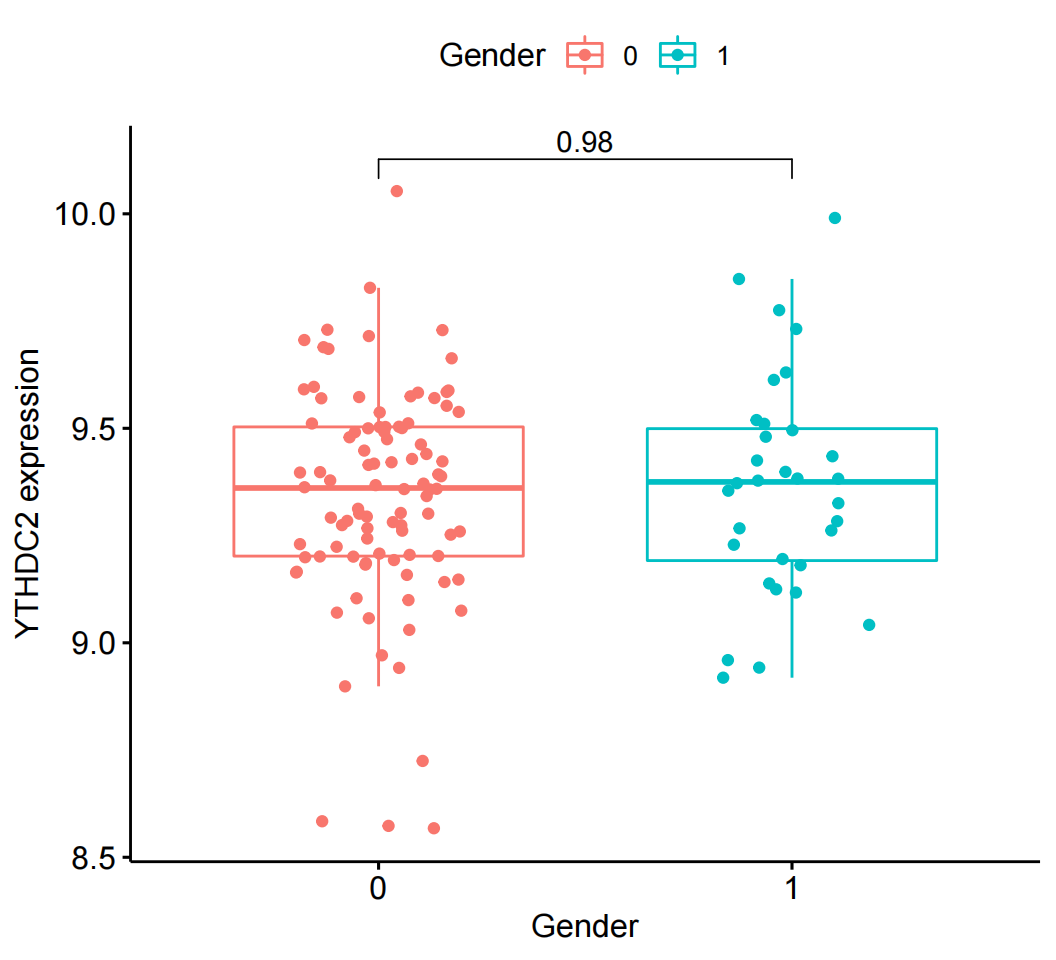


Fig.S1 YTHDC2 expression is independent of age and gender.

| ONTOLOGY | ID | Description | GeneRatio | BgRatio | pvalue | p.adjust | geneID | Count |
| --- | --- | --- | --- | --- | --- | --- | --- | --- |
| BP | GO:  0050792 | regulation of viral process | 19/655 | 162/18903 | 3.55E-06 | 0.010683009 | TRIM31/SRPK2/TARBP2/AXL/CIITA/CXCR4/PPID/VAPB/STAU1/TRIM25/TRIM10/PPIE/VPS4A/IFIT1/UBP1/ATG5/PPIA/TRIM26/DYNLT1 | 19 |
| BP | GO:  0016032 | viral process | 34/655 | 421/18903 | 4.65E-06 | 0.010683009 | TRIM31/SRPK2/PIKFYVE/TARBP2/ST6GAL1/AXL/CIITA/CXCR4/SIGLEC1/MOGS/GYPA/ITGAV/PPID/SNW1/VAPB/STAU1/TRIM25/TRIM10/PPIE/VPS4A/IFIT1/UBP1/CFL1/GTF2B/ATG5/PPIA/CTSB/CAV2/DENR/SLC6A19/ITGB7/TRIM26/DYNLT1/GFI1 | 34 |
| BP | GO:  0045070 | positive regulation of viral genome replication | 8/655 | 32/18903 | 1.00E-05 | 0.012770422 | SRPK2/TARBP2/PPID/VAPB/STAU1/PPIE/IFIT1/PPIA | 8 |
| BP | GO:  0048524 | positive regulation of viral process | 11/655 | 64/18903 | 1.11E-05 | 0.012770422 | SRPK2/TARBP2/AXL/PPID/VAPB/STAU1/PPIE/VPS4A/IFIT1/ATG5/PPIA | 11 |
| BP | GO:  0044000 | movement in host | 19/655 | 183/18903 | 2.08E-05 | 0.019142638 | TRIM31/PIKFYVE/AXL/CIITA/CXCR4/SIGLEC1/GYPA/ITGAV/EXOC7/PPID/TRIM25/TRIM10/VPS4A/PPIA/CTSB/CAV2/ITGB7/TRIM26/DYNLT1 | 19 |
| BP | GO:  0051701 | biological process involved in interaction with host | 20/655 | 203/18903 | 2.74E-05 | 0.020993509 | TRIM31/PIKFYVE/AXL/CIITA/CXCR4/SIGLEC1/GYPA/ITGAV/EXOC7/PPID/TRIM25/TRIM10/VPS4A/IFIT1/PPIA/CTSB/CAV2/ITGB7/TRIM26/DYNLT1 | 20 |
| BP | GO:  0019058 | viral life cycle | 26/655 | 319/18903 | 5.19E-05 | 0.034070402 | TRIM31/SRPK2/PIKFYVE/TARBP2/AXL/CIITA/CXCR4/SIGLEC1/GYPA/ITGAV/PPID/VAPB/STAU1/TRIM25/TRIM10/PPIE/VPS4A/IFIT1/ATG5/PPIA/CTSB/CAV2/SLC6A19/ITGB7/TRIM26/DYNLT1 | 26 |

Tab.S1 GO enrichment results of differential genes in 3 clusters.
